# *dock8* deficiency attenuates microglia colonization in early zebrafish larvae

**DOI:** 10.1038/s41420-022-01155-6

**Published:** 2022-08-17

**Authors:** Linxiu Wu, Rongtao Xue, Jiahao Chen, Jin Xu

**Affiliations:** 1 School of Medicine, South China University of Technology, Guangzhou, Guangdong 510006 China; 2 Department of Hematology, Nanfang Hospital, Southern Medical University, Guangzhou, Guangdong 510515 China

**Keywords:** Development, Microglia

## Abstract

Microglia are tissue-resident macrophages that carry out immune functions in the brain. The deficiency or dysfunction of microglia has been implicated in many neurodegenerative disorders. DOCK8, a member of the DOCK family, functions as a guanine nucleotide exchange factor and plays key roles in immune regulation and neurological diseases. The functions of DOCK8 in microglia development are not fully understood. Here, we generated zebrafish *dock8* mutants by CRISPR/Cas9 genome editing and showed that *dock8* mutations attenuate microglia colonization in the zebrafish midbrain at early larvae stages. In vivo time-lapse imaging revealed that the motility of macrophages was reduced in the *dock8* mutant. We further found that *cdc42/cdc42l*, which encode the small GTPase activated by Dock8, also regulate microglia colonization in zebrafish. Collectively, our study suggests that the Dock8-Cdc42 pathway is required for microglia colonization in zebrafish larvae.

## Introduction

Microglia are tissue-resident macrophages that carry out multiple functions in the central nervous system (CNS) [1, 2]. During development, microglia predominately act as immune cells to remove cellular debris, prune synapses, engulf apoptotic neurons and regulate neuronal activity [3–6]. The deficit or dysfunction of microglia has been implicated in many neurodegenerative disorders, including Alzheimer’s disease, Parkinson’s disease, and Huntington’s disease [7–11].

The term microglia was first used in 1919 and morphological and functional characterization of these cells from development to injury was described by the Spanish neuroscientist Pio del Rio-Hortega [12]. In mice, microglial precursors, namely erythromyeloid precursors (EMPs), are derived from the yolk sac mesoderm (E7.0–E8.5) [13–16]. EMPs generate various types of tissue-resident macrophages, including those moving to the brain to differentiate into microglia (E9.5) [14, 17, 18]. Zebrafish microglia colonization in the brain is remarkably similar to that observed in the mice. The microglia are derived from two sources in zebrafish. Embryonic microglial precursors initiate from the rostral blood island (RBI), the equivalent of mouse YS for myelopoiesis, at 11 h post-fertilization (hpf) [19]. These cells move to the brain at 60 hpf [20]. Adult microglial precursors arise from the ventral wall of the dorsal aorta (VDA) and began to populate the brain from 15 days post-fertilization onward (dpf) [19]. Embryonic microglia of zebrafish are transient and are eventually replaced by adult microglia [21]. At embryonic stages, zebrafish microglial precursors enter the optic tectum of the midbrain by two paths, including the lateral periphery between the eyes and brain and the ventral periphery of the brain in a circulation-independent manner [22]. The Il34-Csf1r pathway could regulate microglial precursor migration to the proximal brain regions [23]. During neuronal apoptosis, lysophosphatidylcholine and nucleotide signaling promote microglial precursor entry into the brain [22, 24]. Slc7a7, a Leu/Arg transporter necessary for microglial brain colonization, has recently been discovered to label a macrophage sub-lineage that migrates to the brain to become microglia [25]. Mobility is a basic property of macrophages. Although factors affecting the mobility of microglia/macrophages have been extensively studied, studies have primarily been carried out in vitro or under pathological conditions [26–28]. How the motility of macrophages affects their brain colonization during animal development is less studied.

Dedicator of cytokinesis 8 (DOCK8) is a member of the evolutionarily conserved DOCK-C subfamily of DOCK family proteins. DOCK8 functions as a CDC42-specific guanine nucleotide exchange factor (GEF) and modulates CDC42 activities [29–31]. DOCK8 has two evolutionarily conserved domains (DHR1 and DHR2) and one functionally uncharacterized domain (DUF3398). DHR1 is a C2 domain found in dedicator of cytokinesis (Dock) class C proteins (C2_Dock-C). This domain binds phosphatidylinositol 4,5-bisphosphate (PI(4,5)P2) to regulate the migration of dendritic cells (DCs) [32]. DHR2, a GEF domain, mediates CDC42 activation to regulate immune cell migration [33–35], control the survival of innate lymphoid cells in the gut [36], and maintain the integrity of lymphocytes [37]. Hence, DOCK8, especially its DHR2 domain, plays a key role in immune regulation. DOCK8 has recently been suggested to function as a signaling adapter to control diverse signaling events in lymphocytes [38]. In humans, DOCK8 deficiency causes a combined immunodeficiency. The symptoms include atopic dermatitis, eczema, recurrent respiratory tract infections, allergies, abscesses, viral infections, and mucocutaneous candidiasis [39, 40]. Interestingly, DOCK8 mutations are associated with neurological disorders, such as CNS vasculitis, stroke and autism [41, 42]. Importantly, DOCK8 deficiency reduced microglial phagocytosis and alleviated neuroinflammation in neurodegenerative disease models suggesting important roles of DOCK8 in microglia under pathological conditions [43]. However, how Dock8 regulates the development of microglia remains unclear.

In this study, we generated *dock8* mutants by CRISPR/Cas9 using zebrafish as a model organism to determine whether *dock8* mutation affects microglia colonization in the zebrafish brain. Our results suggest that *dock8* deficiency attenuates microglia colonization via the DOCK8-Cdc42 pathway by impairing microglia motility in zebrafish early larvae, although the overall the number of macrophages was not affected.

## Results

### *dock8* is expressed in macrophages in zebrafish embryos

To test whether *dock8* could play a role in microglia colonization, we first examined whether *dock8* was expressed in macrophages. Expression of *dock8* was detected by FISH in *Tg(mpeg1:eGFP)* embryos. As shown in Fig. S1, *dock8* positive signals largely overlapped with eGFP^+^ macrophages from 1–2 dpf (Fig. S1D). However, *dock8* was hardly detected in macrophages by FISH after 3 dpf (data not shown). We speculate that the expression of *dock8* in macrophages might decrease as the fish grows. Indeed, we found that *dock8* was expressed in macrophages at 5 dpf by analyzing published single-cell sequencing data, although at a lower level compared with that at 1 dpf (Fig. S1A–C) [44, 45].

### Establishment of the *dock8* mutant

To generate a zebrafish *dock8* mutant, we disrupted the DHR2 domain which is essential for Dock8 function [34]. We first performed multiple sequence alignment for the DHR2 domain using online tools (http://multalin.toulouse.inra.fr/multalin/). As shown in Fig. S2, the DHR2 domain of zebrafish *Dock8* shares over 80% identity with the DHR2 domain of human and mouse. We next designed gRNAs to target exon 39 or exon 45 of the *dock8* gene, where the codons represented conserved amino acids in the DHR2 domain of the *dock8* gene.

**Fig. 1:**
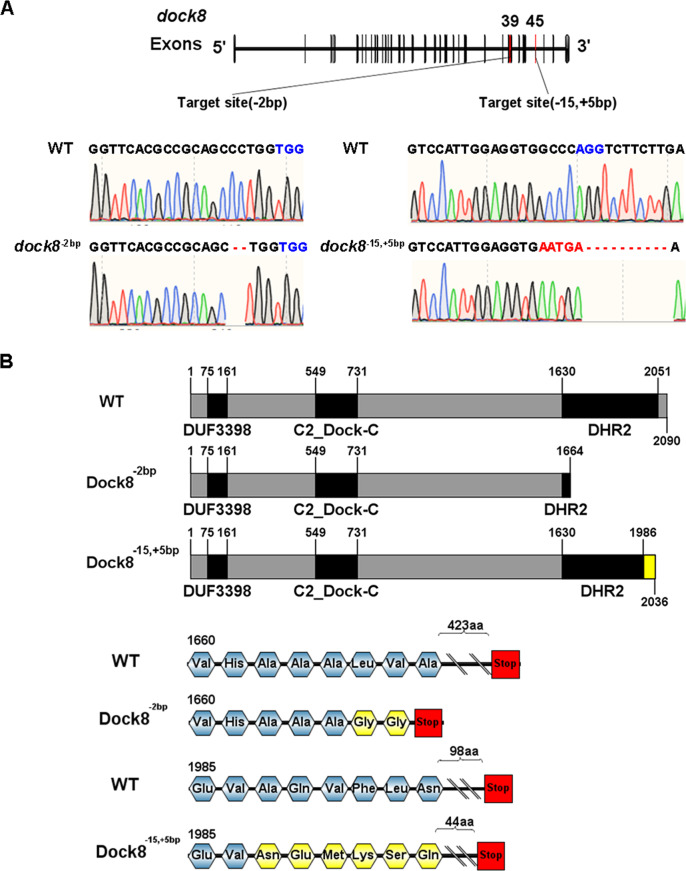
Generation of *dock8*^*-2bp*^ and *dock8*^*-15,+5bp*^ mutants. **A** Sequencing results of the deletion and insertion in *dock8*^*-2bp*^ and *dock8*^*-15,+5bp*^ mutants. Both mutants were generated by CRISPR/Cas9. gRNA targeted at exon 39 and caused 2 bp deletion. gRNA targeted at exon 45 causing 15 bp deletion and 5 bp insertion. PAM sequence was highlighted in blue. Insertion and deletion were highlighted in red. **B** Modular structure of WT and truncated protein. Altered amino acids were labeled in yellow.

We generated *dock8* mutants by co-injection of Cas9 protein and sgRNA into zebrafish embryos at the single-cell stage. Two mutant alleles, with mutations in exon 39 and 45, were recovered. The *dock8*^*−2bp*^ allele lost 2 bp in exon 39 and the *dock8*^*−15, +5bp*^ allele lost 15 bp but gained an extra 5 bp in exon 45 (Fig. 1A). To check whether these two mutant alleles broke the Dock8 protein, we analyzed the codon sequences and found that premature stop codons were introduced in both mutant alleles (Fig. 1B), suggesting possible disruption of Dock8 functions.

### *dock8* deficiency reduces the number of microglia in zebrafish larvae

**Fig. 2:**
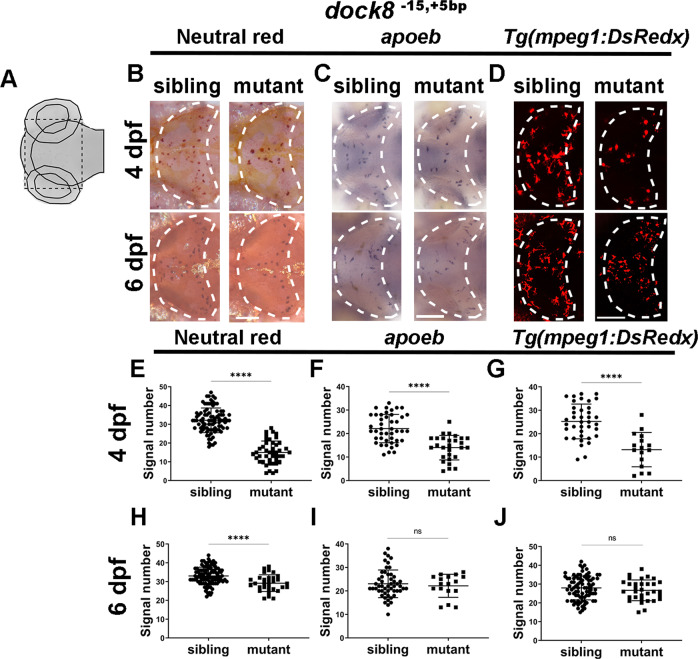
Microglia deficiency in *dock8*^*-15,+5bp*^ mutants. **A** Schematic diagram of the imaging region. Black dash lines represent the imaging area. **B**, **E**, **H** Representative images (**B**) and quantification of 4 dpf (**E**) and 6 dpf (**H**) NR signals of *dock8*^*-15,+5bp*^ mutants and siblings. **C**, **F**, **I** Representative images (**C**) and quantification of 4 dpf (**F**) and 6 dpf (**I**) *apoeb* WISH signals of *dock8*^*-15,+5bp*^ mutants and siblings. **D**, **G**, **J** Representative images (**D**) and quantification of 4 dpf (**G**) and 6 dpf (**J**) mpeg1-dsredx positive cells in optic tectum of *dock8*^*-15,+5bp*^ mutants and siblings. Group sizes were at least *n* = 30 zebrafish embryos. Each dot represents one larva. White dashed lines indicate the optic tectum. Scale bar = 100 µm. Data were analyzed by unpaired Student’s *t*-tests. ^ns^*P* > *0.05*;^****^P ≤ *0.0001*.

To determine whether *dock8* mutation affects microglia colonization, we first investigated the signal of neutral red (NR), which labels microglia lysosomes in the developing brain at 4 dpf when the microglia population has been well established in the brain [46]. The average number of NR signals in *dock8*^*−15,+5bp*^ mutant embryos (~15 cells) was largely reduced compared with that of sibling embryos (~32 cells) (Fig. 2B, E). To further confirm the reduction of microglia in *dock8* mutants, we examined another microglial marker: *apolipoprotein Eb* (*apoeb*) by WISH and the macrophage marker mpeg1 using mpeg1 transgenic fish. A similar reduction in the *apoeb* signal and mpeg1-DsRedx^+^ macrophages/microglia was observed in the brain of *dock8*^*−15,+5bp*^ mutant embryos at 4 dpf (Fig. 2C, D, F, G). Interestingly, macrophages in caudal hematopoietic tissue (CHT) showed no obvious differences between mutants and siblings suggesting that the microglia defect in mutants was not due to an overall reduction of macrophages (Fig. S1E).

To test whether microglia could recover at later developmental stages, we examined NR, *apoeb*, and mpeg1-DsRedx^+^ at 6 dpf. The *apoeb* signal and mpeg1-DsRedx^+^ macrophages/microglia were largely normal in *dock8*^*−15,+5bp*^ mutant embryos suggesting the recovery of microglia (Fig. 2C, D, I, J). Only the NR signal showed slight reduction in mutants (Fig. 2B, H). This could suggest an attenuated function of microglia lysosomes in *dock8*^*−15,+5bp*^ mutants.

**Fig. 3:**
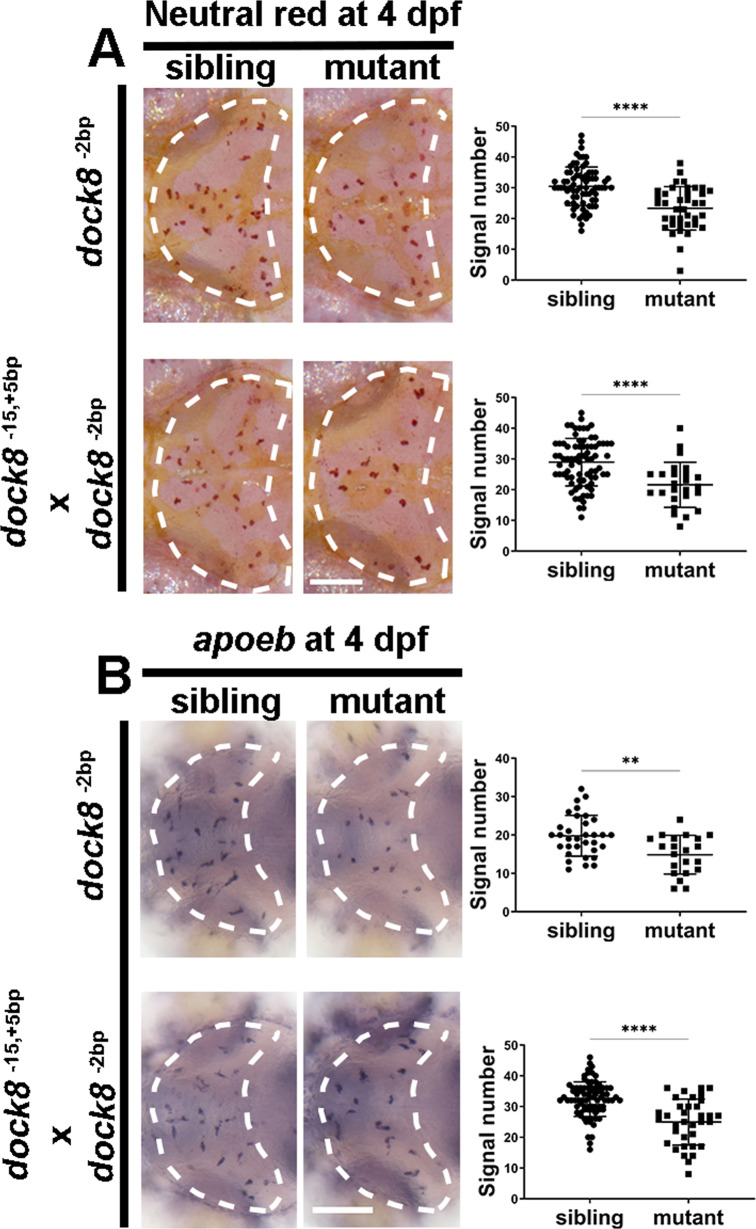
*dock8* is the causative gene of microglia deficiency. **A** Representative images and quantification of 4 dpf NR signals of *dock8*^*-2bp*^ mutants, *dock8*^*-15,+5bp/-2bp*^ compound mutants and siblings. **B** Representative images and quantification of 4 dpf *apoeb* WISH signals of *dock8*^*-2bp*^ mutants, *dock8*^*-15,+5bp/-2bp*^ compound mutants and siblings. Group sizes were at least *n* = 30 zebrafish embryos. Each dot represents one larva. White dashed lines indicate the optic tectum. Scale bar = 100 µm. Data were analyzed by unpaired Student’s *t*-tests. ^**^P ≤ *0.01*; ^****^P ≤ *0.0001*.

To confirm that the reduction of microglia at early developmental stages was indeed caused by the *dock8* deficiency, we performed a rescue experiment. The in vitro-synthesized *dock8* mRNA failed to rescue the *dock8*^*−15,+5bp*^ mutants, likely because the in vitro-synthesized mRNA could not last to 4 dpf. We then examined microglia in *dock8*^*−2bp*^ mutants to determine whether similar defects could be observed in a second mutant allele. Our results showed that both NR and *apoeb* signals were reduced in *dock8*^*−2bp*^ mutants at 4 dpf (Fig. 3). More importantly, similar microglia defects were reproduced in the compound *dock8*^*−15,+5bp/−2bp*^ mutants at 4 dpf (Fig. 3). These results suggest that the reduced microglia are indeed due to *dock8* mutations.

### *dock8* deficiency impairs the motility of macrophages in zebrafish larvae

**Fig. 4:**
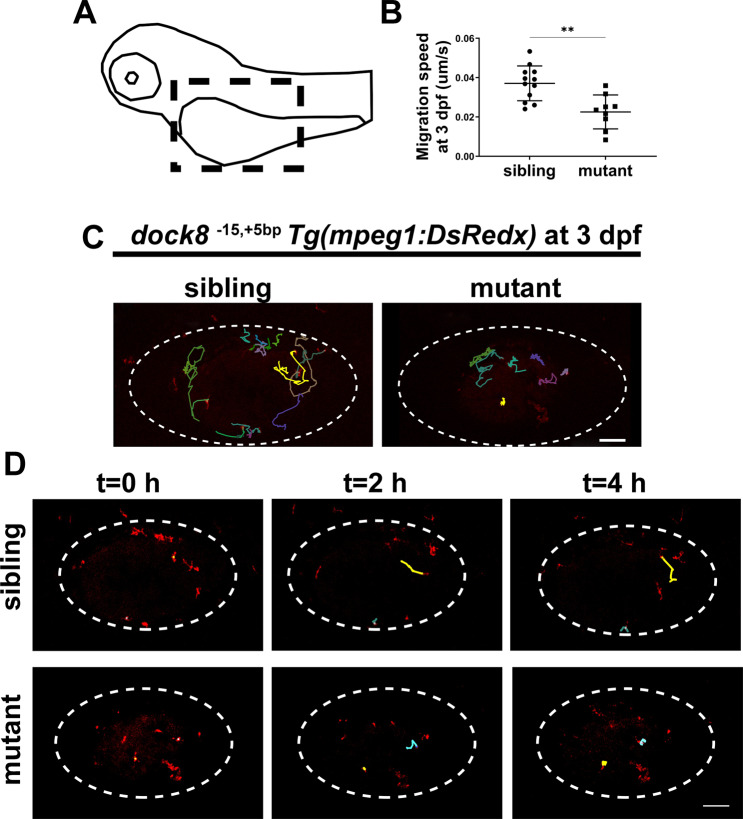
*dock8* deficiency affects migration speed of macrophages in zebrafish larvae. **A** Schematic diagram of the imaging region. Black dash lines represent the imaging area. **B** Quantification of migration speed of mpeg1^+^ cells in *dock8*^*-15,+5bp*^ mutants and siblings yolk sac within 4 h from 3 dpf, each dot represents one larva. **C** Representative images of mpeg1^+^ cells tracking in *dock8*^*-15,+5bp*^ mutants and siblings yolk sac at 3 dpf. Each line represents the migration path of one macrophage. **D** Time-lapse confocal imaging of *Tg(mpeg1:DsRed);dock8*^*15,+5bp*^ embryos from 3 dpf within 4 h. Each line represents the migration path of one macrophage. White dotted lines indicate the yolk sac. Scale bar = 100 µm. See also Video S 1. Data were analyzed by unpaired Student’s *t*-tests. ^**^P ≤ *0.01*.

Some studies have reported that Dock8 regulates macrophage/microglia migration under pathological conditions [33, 43]. We hypothesized that *dock8* deficiency affected microglia colonization by regulating motility of macrophages. To test this hypothesis, we first observed the basic motility of macrophages. Interestingly, we found *dock8* mutant macrophages tend to move more in close range and exhibited slower migration speeds compared with siblings in the yolk sac at 3 dpf (Fig. 4 and Video S 1). These studies indicate that *dock8* deficiency impairs the motility of macrophages and could consequently affect their migration to the brain in zebrafish larvae.

### *dock8* deficiency reduces microglia colonization in early zebrafish larvae

**Fig. 5:**
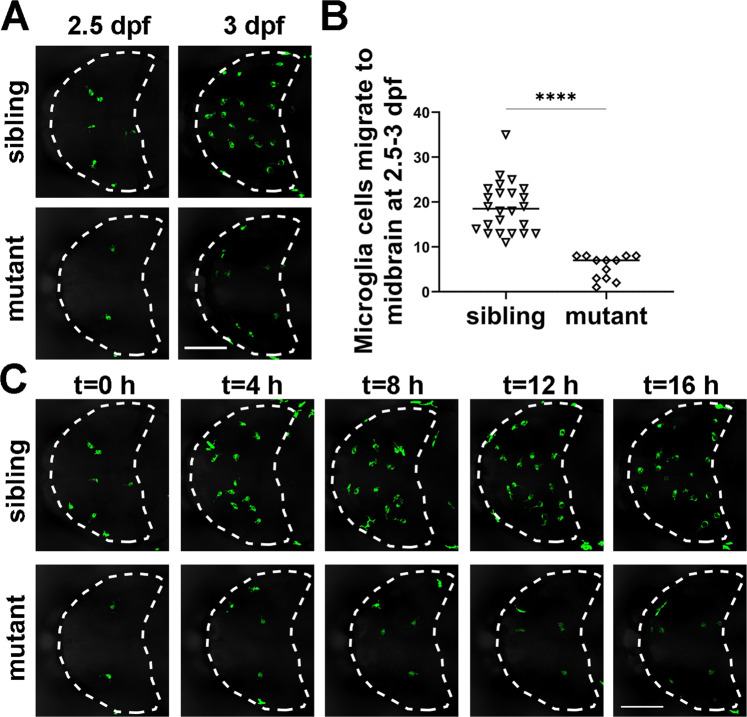
*dock8* deficiency reduce microglia colonization in zebrafish early larvae. **A** Representative images of GFP^+^ microglia in *dock8*^*-15,+5bp*^; *Tg(coro1a:GFP)* embryos from 2.5 dpf–3 dpf for 10–16 h. **B** Quantification of GFP^+^ microglia migrating into the midbrain from 2.5 dpf to 3 dpf in *dock8*^*-15,+5bp*^*; Tg(coro1a:GFP)* embryos. **C** Time-lapse confocal imaging of microglia colonization in *dock8*^*-15,+5bp*^; *Tg(coro1a:GFP)* embryos from 2.5 dpf–3 dpf. Each dot represents one larva. White dotted lines indicate the midbrain. Scale bar = 100 µm. See also Video S2. Data were analyzed by unpaired Student’s *t*-tests. ^****^P ≤ *0.0001*.

To confirm that impaired macrophage motility of *dock*8 mutants leads to microglia paucity at early developmental stages, we directly monitored the colonization of microglia from 2.5–3 dpf by live imaging. As shown in Fig. 5 and Video S 2, the average number of GFP positive microglia migrating into the brain in *dock8*^*−15,+5bp*^ mutant embryos was significantly less than those in sibling embryos. These studies suggest that *dock8* deficiency reduces microglia colonization by impairing its motility in early zebrafish larvae.

### *cdc42* acts downstream of *dock8* to affect microglia colonization in zebrafish larvae

Dock8 is known to mediate Cdc42 activation and subsequent migration of DCs, T cells, and macrophages [33–35]. We then tested whether microglia colonization is regulated by the DOCK8-CDC42 module in zebrafish. There are three *cdc42* homologs, *cdc42*, *cdc42l*, and *cdc42l2*, in zebrafish. Interestingly, analysis of published single-cell sequencing data showed that only *cdc42* and *cdc42l* were readily detected in embryonic macrophages (Fig. S3) [44, 45]. We hypothesized that *cdc42* and *cdc42l*, but not *cdc42l2*, could be involved in macrophages. Therefore, we generated deletions of exon 3 of *cdc42* and of exon 2 of *cdc42l*. The resulting *cdc42* mutant lost 20 bp and the *cdc42l* mutant lost 29 bp. These deletions resulted in a premature stop codon in Cdc42 and Cdc42l (Fig. S4).

**Fig. 6:**
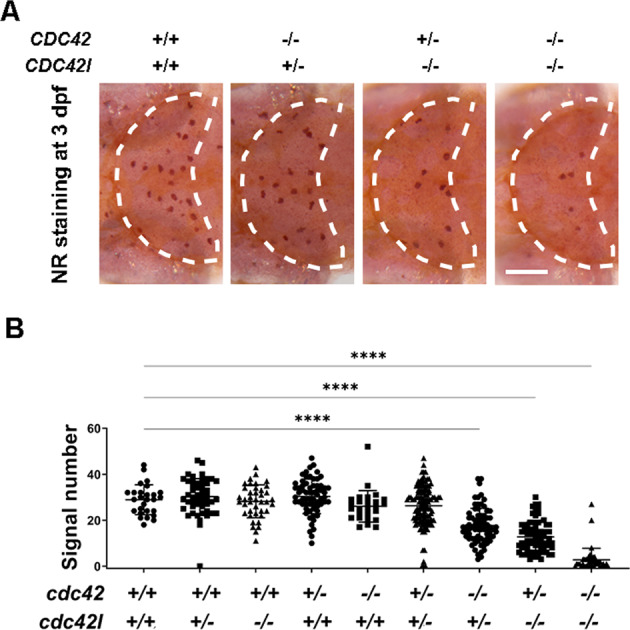
*dock8* affect microglia colonization in zebrafish early larvae via *cdc42*. **A** Representative image of NR staining in *cdc42*^*-20bp*^*cdc42l*^*−29bp*^ double mutants at 3 dpf. **B** Quantification of NR signals in *cdc42*^*−20bp*^*cdc42l*^*−29bp*^ double mutants at 3 dpf. Group sizes were at least *n* = 48 zebrafish embryos. Each dot represents one larva. White dashed lines indicate the optic tectum. Scale bar = 100 µm. Data were analyzed by one-way ANOVA followed by Dunnett’s multiple comparisons test. ^****^P ≤ *0.0001*.

To determine whether *cdc42* or *cdc42l* affected microglia colonization, we investigated the NR signal in these mutants. NR signals in *cdc42*^-/-^ or *cdc42l*^*-/-*^ single mutants were similar to wild-type embryos at 3 dpf suggesting that the number of microglia was normal in single mutants (Fig. 6B). Interestingly, NR signals in *cdc42*^*-/-*^*cdc42l*^*+/-*^ (~17.66 cells), *cdc42*^*+/-*^*cdc42l*^*-/-*^ (~12.72 cells), and *cdc42l*^*-/-*^*cdc42l*^*-/-*^ embryos (~2.7 cells) were significantly reduced compared with that of double wild-type embryos (~28.89 cells) (Fig. 6). We further confirm a reduction of microglia in *cdc42* and *cdc42l* mutants by WISH (Fig. S5). A reduction of the *mpeg1* signal was observed in the brains of *cdc42*^*-/-*^*cdc42l*^*+/+*^*, cdc42*^*-/-*^*cdc42l*^*+/-*^, *cdc42*^*+/-*^*cdc42l*^*-/-*^, and *cdc42l*^*-/-*^*cdc42l*^*-/-*^ embryos. By contrast, there were no obvious differences between mutant and sibling macrophages in the body (Fig. S5), suggesting that the microglia defect in the mutants was not due to an overall reduction of macrophages. We noticed that *cdc42*^*-/-*^*cdc42l*^*+/+*^ only showed a slight reduction of *mpeg1* signals but not NR staining. This could have been a result of different staining methods or different batches of fish. Taken together, these results suggest that *cdc42* and *cdc42l* redundantly regulate early microglia colonization.

## Discussion

In this study, we reveal that *dock8* mutation affects microglia colonization in zebrafish early larvae. We further showed that *cdc42/cdc42l* mutants have similar defects in microglia colonization suggesting that the DOCK8-CDC42 pathway regulates microglia colonization. Until now, little has been known of the factors upstream of Dock8 to regulate microglia colonization. The Il34-Csf1r pathway has been reported to regulate the migration and colonization of microglial precursors in zebrafish [23]. Interestingly, CSF-1, a ligand of CSF1r, activates Rac and Cdc42 [47]. In addition, Vav, as a Rac-GEF, is activated by CSF-1 to participate in cytoskeletal changes in osteoclasts [47]. Importantly, Cdc42 regulates CSF-1-induced polarization of macrophages [48]. It would be interesting to explore whether Csf1r functions upstream of Dock8 to regulate the migration and colonization of microglial precursors.

Our investigations revealed that *dock8* deficiency impaired microglia and/or macrophage motility through the DOCK8-CDC42 pathway. However, how DOCK8 regulates CDC42 to affect these processes remains unclear. Interestingly, Cdc42, via DOCK8, binds effector molecules, such as myotonic dystrophy kinase-related Cdc42-binding kinase (MRCK), through facilitated myosin II (MLC2) phosphorylation to regulate macrophage migration [33]. In addition, CDC42-GTP generated by DOCK8 binds WASp to regulate actin polymerization, T cell transendothelial migration, and homing to lymph nodes [49]. Therefore, we speculate that DOCK8 might regulate CDC42 to affect migration colonization through the above mechanisms.

Our results show a 50% reduction in microglia numbers in *dock8* mutants at 4 dpf, but the number of peripheral macrophages was not affected, suggesting *dock8* is not indispensable for early macrophage development. Interestingly, the recovery of microglia numbers in *dock8* mutants suggests that the colonization of microglia is only temporarily affected. This result suggests that complementary machinery exists to promote the colonization of microglia in *dock8* mutants. We speculate that other DOCK family proteins could play important roles, as Dock proteins are known to regulate the actin cytoskeleton, cell adhesion and migration [50]. Recent reports have suggested that DOCK10 and DOCK11 function as Cdc42 and/or Rac1 GEFs to modulate the mobility of macrophages and/or microglia [51, 52]. Therefore, DOCK8 could work together with other DOCK family members to regulate microglia colonization. We also noticed that the phenotype of microglia colonization is more severe in the *cdc42/cdc42l* double mutant compared with the *dock8* mutant. One possible reason is that other DOCK proteins, such as DOCK10 and DOCK11 [52], function in parallel with DOCK8 to modulate CDC42 activities together. Another possibility is there are other signaling events to activate CDC42. Further studies are warranted to reveal other machineries in addition to the Dock8-Cdc42 pathway.

## Materials and methods

### Zebrafish husbandry

All zebrafish were raised and bred at 28.5°C in a standard circulating water system and embryos were incubated at the same temperature in incubators. Embryos were maintained in egg water (E2 with methylene blue) with or without 0.003% N-phenylthiourea (PTU; Sigma Aldrich) to avoid pigmentation [53]. The following mutants and transgenic lines were used in this study: AB, *dock8*^*szy104*^ (abbreviated as *dock8*^*-15,+5bp*^) mutant, *dock8*^*szy105*^ (abbreviated as *dock8*^*-2bp*^) mutant, *cdc42*^*szy106*^ (abbreviated as *cdc42*^*-20bp*^) mutant, *cdc42l*^*szy107*^ (abbreviated as *cdc42l*^*-29bp*^) mutant, *Tg(mpeg1:loxP-DsRedx-loxP-GFP)*^*hkz15Tg*^ (abbreviated as *Tg(mpeg1:DsRedx)*) [54], *Tg(mpeg1:GFP)* [55], and *Tg(coro1a:GFP)* [19].

### Generation of zebrafish mutants

The sgRNA sequence of the *dock8*, *cdc42*, and *cdc42l* genes were designed according to the recommended protocol [56–59]. The *dock8*^*-2bp*^ sgRNA (5′-ggttcacgccgcagccctgg-3′), the *dock8*^*-15,+5bp*^ sgRNA (5′-gggtccattggaggtggccc-3′), the *cdc42*^*-20bp*^ sgRNA (5′-ggtggtgagccgtacaccct-3′), the *cdc42l*^*-29bp*^ sgRNA (5′-gaggggaaccgtatacactg-3′) were synthesized using T7 RNA polymerase (Thermo Fisher Scientific, USA). Cas9 protein and sgRNA were co-injected into zebrafish embryos at the single-cell stage. Cas9 efficiency was assessed by T7E1 (New England Biolabs) digestion of the PCR product of genomic DNA from embryos injection at 24 hpf.

### Sequencing and genotyping

Genomic DNA was harvested from clipped tails. Primers used for PCR of genomic DNA were as follows: *dock8*^*-2bp*^-fwd: 5′-accgagtggcacatttgagc-3′, *dock8*^*-2bp*^-rev: 5′-gagacataattcacgcctac-3′; *dock8*^*-15,+5bp*^-fwd: 5′-tggcactgagatgagacacc-3′, *dock8*^*-15,+5bp*^-rev: 5′-ggttgtccttcatctacgtg-3′; *cdc42*^*-20bp*^-fwd: 5′-catgccatgacctgtctcgt-3′, *cdc42*^*-20bp*^-rev: 5′-cagtacctgcagtatcaaac-3′; *cdc42l*^*-29bp*^-fwd: 5′-ctaatgtgtgacgtccagtg-3′, *cdc42l*^*-29bp*^-rev: 5′-actggctatacacatcagac-3′. Genotypes of *dock8*^*-2bp*^ or *dock8*^*-15,+5bp*^ mutants were determined by BsaJI digestion of the PCR products. Genotypes of *cdc42*^*-20bp*^ or *cdc42l*^*-29bp*^ mutants were determined according to the size difference of PCR products between mutants and wild-type embryos.

### Neutral red staining

Neutral red solution (Cat#G1315, Solarbio) was diluted 1:1000 in egg water with 0.003% PTU. Zebrafish embryos were incubated in this solution for 5–6 h at 28.5°C in the dark. Images were taken with a fluorescence stereomicroscope (zoom V16) using a 100x objective. Quantification was performed manually.

### In vitro synthesis of antisense RNA probe, whole-mount in situ hybridization, and antibody staining

Antisense DIG labeled RNA probe of *dock8* was synthesized in vitro according to the standard protocol. Primers for *dock8* probe synthesis were as follows:

XmaI-FP: ctgcagcccgggtcatgcaggagttgggtgag,

XbaI-RP: ggccgctctagatcacagcatccggtttctcc.

The *apoeb* probe was described previously [20, 60]. The *mpeg1* probe was a gift from Professor Zilong Wen of Hong Kong University of Science and Technology. Whole mount in situ (WISH) was performed as described previously [61] with NBT/BCIP (11697471001, Roche) or BM purple AP (11442074001, Roche) or TSA^TM^ Plus Cyanine 3 System (NEL744001KT, Perkin-Elmer). Images were captured with a fluorescence stereomicroscope (zoom V16) using a 100x or 40x objective. Quantification was performed manually.

Antibody staining was performed as previously reported [62]. The primary antibody was a goat-anti-GFP antibody (ab6658, Abcam;1:400). The secondary antibody is donkey anti-Goat IgG, Alexa Fluor^TM^ 488 antibody (A11055 Invitrogen; 1:400). Images were captured with a Zeiss LSM 800 confocal microscope using a 10x or 20x objective.

### Time-lapse imaging and cell tracking analysis

Time-lapse imaging was performed as previously reported with some modifications [22]. Embryos were anesthetized in 0.01% tricaine (A5040; Sigma), mounted in 1% low-melting agarose and covered with egg water (0.01% tricaine and PTU). Then embryos were imaged on a Zeiss LSM 880 confocal microscope with a 10x or 20x objective in a 28.5 °C thermal chamber. Z-step size was set at 3 µm; usually, 28–30 Z steps for embryos at 2.5–3 dpf or 13–18 Z steps for embryos at 3 dpf are used at 2–3 min intervals. The total observation time was 12–16 h for the fish brain or 4 h for the fish yolk using the 488 nm and/or 561 nm laser. Images were processed with Zeiss ZEN or Imaris software.

### Statistical analysis

All statistical analysis was performed using GraphPad Prism version 9.0 with unpaired Student’s *t*-tests and one-way ANOVA followed by Dunnett’s multiple comparisons test of more than two samples to calculate significance. Asterisks indicate statistical differences (*p ≤ *0.05*, **p ≤ *0.01*, ***p ≤ *0.001*, ****p  ≤*0.0001*). Data were collected from at least three independent experiments.

## Supplementary information

The information of the supplementary figure legends

dock8 is expressed in macrophages in early larval stage

Amino acid alignment of the DHR2 domain in Dock8 from human, mouse, zebrafish and dock8 mutant

cdc42, cdc42l and cdc42l2 expression in macrophages

Generation of the cdc42 and cdc42l mutants by CRISPR/Cas9

Microglia deficiency in cdc42 and cdc42l mutants

dock8 deficiency impairs the migration speed of macrophage in zebrafish larvae

dock8 deficiency reduces microglia colonization in zebrafish early larvae

## Data Availability

The datasets used and/or analyzed that support the findings of this study are available from the corresponding author upon request.

## References

[1] Streit WJ, Graeber MB, Kreutzberg GW (1988). Functional plasticity of microglia: a review. Glia.

[2] Aguzzi A, Barres BA, Bennett ML (2013). Microglia: Scapegoat, saboteur, or something else?. Science.

[3] Herzog C, Garcia LP, Keatinge M, Greenald D, Moritz C, Peri F (2019). Rapid clearance of cellular debris by microglia limits secondary neuronal cell death after brain injury in vivo. Development..

[4] Paolicelli RC, Bolasco G, Pagani F, Maggi L, Scianni M, Panzanelli P (2011). Synaptic pruning by microglia is necessary for normal brain development. Science.

[5] Stolzing A, Grune T (2004). Neuronal apoptotic bodies: phagocytosis and degradation by primary microglial cells. FASEB J.

[6] Li Y, Du X, Liu C, Wen Z, Du J (2012). Reciprocal regulation between resting microglial dynamics and neuronal activity in vivo. Dev. Cell.

[7] McGeer PL, McGeer EG (1995). The inflammatory response system of brain: implications for therapy of Alzheimer and other neurodegenerative diseases. Brain Res Brain Res Rev.

[8] Onuska KM (2020). The dual role of microglia in the progression of Alzheimer’s disease. J Neurosci.

[9] Ho MS (2019). Microglia in Parkinson’s disease. Adv Exp Med Biol.

[10] Tai YF, Pavese N, Gerhard A, Tabrizi SJ, Barker RA, Brooks DJ (2007). Microglial activation in presymptomatic Huntington’s disease gene carriers. Brain.

[11] Brettschneider J, Toledo JB, van Deerlin VM, Elman L, McCluskey L, Lee VMY (2012). Microglial activation correlates with disease progression and upper motor neuron clinical symptoms in amyotrophic lateral sclerosis. PLoS One.

[12] Del Rio-Hortega P (1919). El "tercer elemento" de los centros nerviosos, poder fagocitario y movilidad de la microglía. I. La microglia en estado normal. Bol de la Soc Española de Biol SC.

[13] Du X, Du J (2016). A death trap for microglia. Dev cell.

[14] Ginhoux F, Greter M, Leboeuf M, Nandi S, See P, Gokhan S (2010). Fate mapping analysis reveals that adult microglia derive from primitive macrophages. Science.

[15] Gomez Perdiguero E, Klapproth K, Schulz C, Busch K, Azzoni E, Crozet L (2015). Tissue-resident macrophages originate from yolk-sac-derived erythro-myeloid progenitors. Nature.

[16] Kierdorf K, Erny D, Goldmann T, Sander V, Schulz C, Perdiguero EG (2013). Microglia emerge from erythromyeloid precursors via Pu.1- and Irf8-dependent pathways. Nat Neurosci.

[17] Li Q, Barres BA (2018). Microglia and macrophages in brain homeostasis and disease. Nat Rev Immunol.

[18] Hoeffel G, Chen J, Lavin Y, Low D, Almeida Francisca F, See P (2015). C-myb^+^ erythro-myeloid progenitor-derived fetal monocytes give rise to adult tissue-resident macrophages. Immun (Camb, Mass).

[19] Xu J, Zhu L, He S, Wu Y, Jin W, Yu T (2015). Temporal-spatial resolution fate mapping reveals distinct origins for embryonic and adult microglia in zebrafish. Dev Cell.

[20] Herbomel P, Thisse B, Thisse C (2001). Zebrafish early macrophages colonize cephalic mesenchyme and developing brain, retina, and epidermis through a m-csf receptor-dependent invasive process. Dev Biol.

[21] Ferrero G, Mahony CB, Dupuis E, Yvernogeau L, Di Ruggiero E, Miserocchi M (2018). Embryonic microglia derive from primitive macrophages and are replaced by cmyb-dependent definitive microglia in zebrafish. Cell Rep. (Camb).

[22] Xu J, Wang T, Wu Y, Jin W, Wen Z (2016). Microglia colonization of developing zebrafish midbrain is promoted by apoptotic neuron and lysophosphatidylcholine. Dev Cell.

[23] Wu S, Xue R, Hassan S, Nguyen TML, Wang T, Pan H (2018). Il34-Csf1r pathway regulates the migration and colonization of microglial precursors. Dev Cell.

[24] Casano Alessandra M, Albert M, Peri F (2016). Developmental apoptosis mediates entry and positioning of microglia in the zebrafish brain. Cell Rep. (Camb).

[25] Rossi F, Casano Alessandra M, Henke K, Richter K, Peri F (2015). The SlC7A7 transporter identifies microglial precursors prior to entry into the brain. Cell Rep. (Camb).

[26] Smolders SM-T, Kessels S, Vangansewinkel T, Rigo J-M, Legendre P, Brône B (2019). Microglia: Brain cells on the move. Prog Neurobiol.

[27] Garden GA, Möller T (2006). Microglia biology in health and disease. J Neuroimmune Pharmacol.

[28] Cardona AE, Li M, Liu L, Savarin C, Ransohoff RM (2008). Chemokines in and out of the central nervous system: much more than chemotaxis and inflammation. J Leukoc Biol.

[29] Cote JF, Vuori K (2002). Identification of an evolutionarily conserved superfamily of DOCK180-related proteins with guanine nucleotide exchange activity. J Cell Sci.

[30] Ruusala A, Aspenstrm P (2004). Isolation and characterisation of DOCK8, a member of the DOCK180-related regulators of cell morphology. Febs Lett.

[31] Kunimura K, Uruno T, Fukui Y (2020). DOCK-family proteins: key players in immune surveillance mechanisms. Int Immunol.

[32] Sakurai T, Kukimoto-Niino M, Kunimura K, Yamane N, Sakata D, Aihara R (2021). A conserved PI(4,5)P2-binding domain is critical for immune regulatory function of DOCK8. Life Sci Alliance.

[33] Shiraishi A, Uruno T, Sanematsu F, Ushijima M, Sakata D, Hara T (2017). DOCK8 protein regulates macrophage migration through Cdc42 protein activation and LRAP35a protein interaction. J Biol Chem.

[34] Harada Y, Tanaka Y, Terasawa M, Pieczyk M, Habiro K, Katakai T (2012). DOCK8 is a Cdc42 activator critical for interstitial dendritic cell migration during immune responses. Blood.

[35] Xu X, Lei H, Zhao G, Xue S, Wang H (2016). LRCH1 interferes with DOCK8-Cdc42-induced T cell migration and ameliorates experimental autoimmune encephalomyelitis. J Exp Med.

[36] Aihara R, Kunimura K, Watanabe M, Uruno T, Yamane N, Sakurai T (2021). DOCK8 controls survival of group 3 innate lymphoid cells in the gut through Cdc42 activation. Int Immunol.

[37] Zhang Q, Dove CG, Hor JL, Murdock HM, Strauss-Albee DM, Garcia JA (2014). DOCK8 regulates lymphocyte shape integrity for skin antiviral immunity. J Exp Med.

[38] Kearney CJ, Randall KL, Oliaro J (2017). DOCK8 regulates signal transduction events to control immunity. Cell Mol Immunol.

[39] Aydin SE, Kilic SS, Aytekin C, Kumar A, Porras O, Kainulainen L (2015). DOCK8 Deficiency: Clinical and immunological phenotype and treatment options-a review of 136 patients. J Clin Immunol.

[40] Zhang Q, Davis JC, Dove CG, Su HC (2010). Genetic, clinical, and laboratory markers for DOCK8 immunodeficiency syndrome. Dis Markers.

[41] AlKhater SA (2016). CNS vasculitis and stroke as a complication of DOCK8 deficiency: a case report. BMC Neurol.

[42] Vinci G, Chantot-Bastaraud S, El Houate B, Lortat-Jacob S, Brauner R, McElreavey K (2007). Association of deletion 9p, 46,XY gonadal dysgenesis and autistic spectrum disorder. Mol Hum Reprod.

[43] Namekata K, Guo XL, Kimura A, Arai N, Harada C, Harada T (2019). DOCK8 is expressed in microglia, and it regulates microglial activity during neurodegeneration in murine disease models. J Biol Chem.

[44] Speir ML, Bhaduri A, Markov NS, Moreno P, Nowakowski TJ, Papatheodorou I (2021). UCSC Cell Browser: visualize your single-cell data. Bioinformatics.

[45] Farnsworth DR, Saunders LM, Miller AC (2020). A single-cell transcriptome atlas for zebrafish development. Dev Biol.

[46] Jin W, Dai Y, Li F, Zhu L, Huang Z, Liu W (2019). Dysregulation of microglial function contributes to neuronal impairment in mcoln1a-deficient zebrafish. iScience.

[47] Sakai H, Chen Y, Itokawa T, Yu K, Zhu M, Insogna K (2006). Activated c-Fms recruits Vav and Rac during CSF-1-induced cytoskeletal remodeling and spreading in osteoclasts. Bone.

[48] Allen WE, Zicha D, Ridley AJ, Jones GE (1998). A role for Cdc42 in macrophage chemotaxis. J Cell Biol.

[49] Janssen E, Tohme M, Hedayat M, Leick M, Kumari S, Ramesh N (2016). A DOCK8-WIP-WASp complex links T cell receptors to the actin cytoskeleton. J Clin Investig.

[50] Gadea G, Blangy A (2014). Dock-family exchange factors in cell migration and disease. Eur J Cell Biol.

[51] Ruiz-Lafuente N, Alcaraz-García M-J, García-Serna A-M, Sebastián-Ruiz S, Moya-Quiles M-R, García-Alonso A-M (2015). Dock10, a Cdc42 and Rac1 GEF, induces loss of elongation, filopodia, and ruffles in cervical cancer epithelial HeLa cells. Biol Open.

[52] Namekata K, Guo X, Kimura A, Azuchi Y, Kitamura Y, Harada C (2020). Roles of the DOCK-D family proteins in a mouse model of neuroinflammation. J Biol Chem.

[53] Westerfield M. The zebrafish book: a guide for the laboratory use of zebrafish (Danio rerio) 3rd edn. *University of Oregon Press*. 2000.

[54] He S, Chen J, Jiang Y, Wu Y, Zhu L, Jin W (2018). Adult zebrafish langerhans cells arise from hematopoietic stem/progenitor cells. eLife.

[55] Yu T, Guo W, Tian Y, Xu J, Chen J, Li L (2017). Distinct regulatory networks control the development of macrophages of different origins in zebrafish. Blood.

[56] Haeussler M, Schönig K, Eckert H, Eschstruth A, Mianné J, Renaud J-B (2016). Evaluation of off-target and on-target scoring algorithms and integration into the guide RNA selection tool CRISPOR. Genome Biol.

[57] Concordet J-P, Haeussler M (2018). CRISPOR: intuitive guide selection for CRISPR/Cas9 genome editing experiments and screens. Nucleic Acids Res.

[58] Labun K, Montague TG, Krause M, Torres Cleuren YN, Tjeldnes H, Valen E (2019). CHOPCHOP v3: expanding the CRISPR web toolbox beyond genome editing. Nucleic Acids Res.

[59] Stemmer M, Thumberger T, Keyer MDS, Wittbrodt J, Mateo JL (2017). Cctop: An intuitive, flexible and reliable Crispr/Cas9 target prediction tool (vol 10, e0124633, 2015). PLoS One.

[60] Babin PJ, Thisse C, Durliat M, Andre M, Akimenko M-A, Thisse B (1997). Both apolipoprotein e and a-i genes are present in a nonmammalian vertebrate and are highly expressed during embryonic development. Proc Natl Acad Sci USA.

[61] Thisse C, Thisse B (2008). High-resolution in situ hybridization to whole-mount zebrafish embryos. Nat Protoc.

[62] Lin X, Zhou Q, Zhao C, Lin G, Xu J, Wen Z (2019). An ectoderm-derived myeloid-like cell population functions as antigen transporters for langerhans cells in zebrafish epidermis. Dev Cell.

