# Supplementary material for: dock8 deficiency attenuates microglia colonization in early zebrafish larvae
Source: Cell Death Discov. 2022 Aug 17;8:366. doi: 10.1038/s41420-022-01155-6 (PMC9386030; doi:10.1038/s41420-022-01155-6)
Supplement: Supplementary file 1 — The information of the supplementary figure legends [file 41420_2022_1155_MOESM1_ESM.doc]

**Fig. S1 *dock8* is expressed in macrophages in early larval stage.**

**A-C** Single cell sequence data from the UCSC browser indicates *dock8* expression in macrophage clusters. (**A**) *dock8* expression profile in macrophage cluster 71 and 184. a and b representing two independent sequence samples. (**B**) *dock8* expression level in macrophages at 1, 2 and 5 dpf (those outlined in black in the edge of the cell). (**C**) Quantification of the expression level of *dock8* in single cell at 1, 2 and 5 dpf. (**D**) *dock8* expression in *Tg(mpeg1:GFP)* at 1 dpf, 1.5 dpf and 2 dpf. A dig-labeled *dock8* probe were used to detect the expression of *dock8* (red). Macrophages were labeled with anti-eGFP antibody (green). White arrowheads indicate *mpeg1^+^* and *dock8^+^* cells. (**E**) Peripheral macrophages development was not affected in *dock8^-15,+5bp^ Tg(mpeg1:DsRed)* mutant embryos at 4 dpf. Group sizes were at least n=30 zebrafish embryos. Each dot represents one larva. White dotted lines indicate the CHT. Scale bar = 100 µm, S1C data were analyzed by one-way ANOVA followed by Dunnett’s multiple comparisons test, S1E data were analyzed by unpaired Student’s t tests. *^*^P ≤ 0.05,* ^ns^*P＞0.05*.

**Fig. S2 Amino acid alignment of the DHR2 domain in Dock8 from human, mouse, zebrafish and *dock8* mutant.**

Highlighted red characters show high consensus in five group and blue frames indicates similarity across groups. Black characters show low similarity across groups. The frameshift in the loss-of-function allele is marked with dots.

**Fig. S3 *cdc42*, *cdc42l* and *cdc42l2* expression in macrophages.**

Expression profiles of *cdc42*, *cdc42l* and *cdc42l2* in macrophages clusters. The data is from the published single cell sequencing data in UCSC Cell Browser.

**Fig. S4 Generation of the *cdc42 and cdc42l* mutants by CRISPR/Cas9.**

**A** Sequencing result of the deletion in *cdc42* mutants. gRNA targeted at exon 3 and caused 20 bp deletion. **B** Sequencing results of the deletion in *cdc42l* mutants. gRNA targeted at exon 2 and caused 29 bp deletion. Deleted nucleotides were highlighted in red. **C** Modular structure of WT and truncated protein in *cdc42* mutants. Altered amino acids were labeled in yellow. **D** Modular structure of WT and truncated protein in *cdc42l* mutants. Altered amino acids were labeled in yellow.

**Fig. S5 Microglia deficiency in *cdc42 and c4c42l* mutants.**

**A** Representative image of *mpeg1* WISH in *cdc42^+/+^cdc42l^+/+^*, *cdc42^-/-^cdc42l^+/-^*, *cdc42^+/-^cdc42l^-/-^* and *cdc42l^-/-^cdc42l^-/-^* embryos at 3 dpf. **B** Quantification of *mpeg1* signals in brain. **C** Quantification of *mpeg1* signals in body. Group sizes were at least n=50 zebrafish embryos. Each dot represents one larva. Black dashed lines indicate the counting region. White dashed lines indicate the optic tectum. Scale bar = 100 µm. Data were analyzed by one-way ANOVA followed by Dunnett’s multiple comparisons test. *^*^P ≤ 0.05; ^***^P ≤ 0.001; ^****^P ≤ 0.0001*.

**Video S1. *dock8* deficiency impairs the migration speed of macrophage in zebrafish larvae.**

Confocal time-lapse imaging showed the macrophage tracking at 3 dpf for 4 hours on the yolk sac. Images were taken with a Zeiss LSM 880 confocal microscope, using a 10x objective. White dotted lines indicate the imaging region. Scale bar = 100 µm. Related to Fig. 4.

**Video S2. *dock8* deficiency reduces microglia colonization in zebrafish early larvae.**

Confocal time-lapse imaging showed microglia migrating into midbrain at 2.5 dpf to 3 dpf. Images were taken with a Zeiss LSM 880 confocal microscope, using a 20x objective. White dotted lines indicate the midbrain. Scale bar = 100 µm. Related to Fig. 5.
