# Supplementary figures and images for: dock8 deficiency attenuates microglia colonization in early zebrafish larvae
Source: Cell Death Discov. 2022 Aug 17;8:366. doi: 10.1038/s41420-022-01155-6 (PMC9386030; doi:10.1038/s41420-022-01155-6)

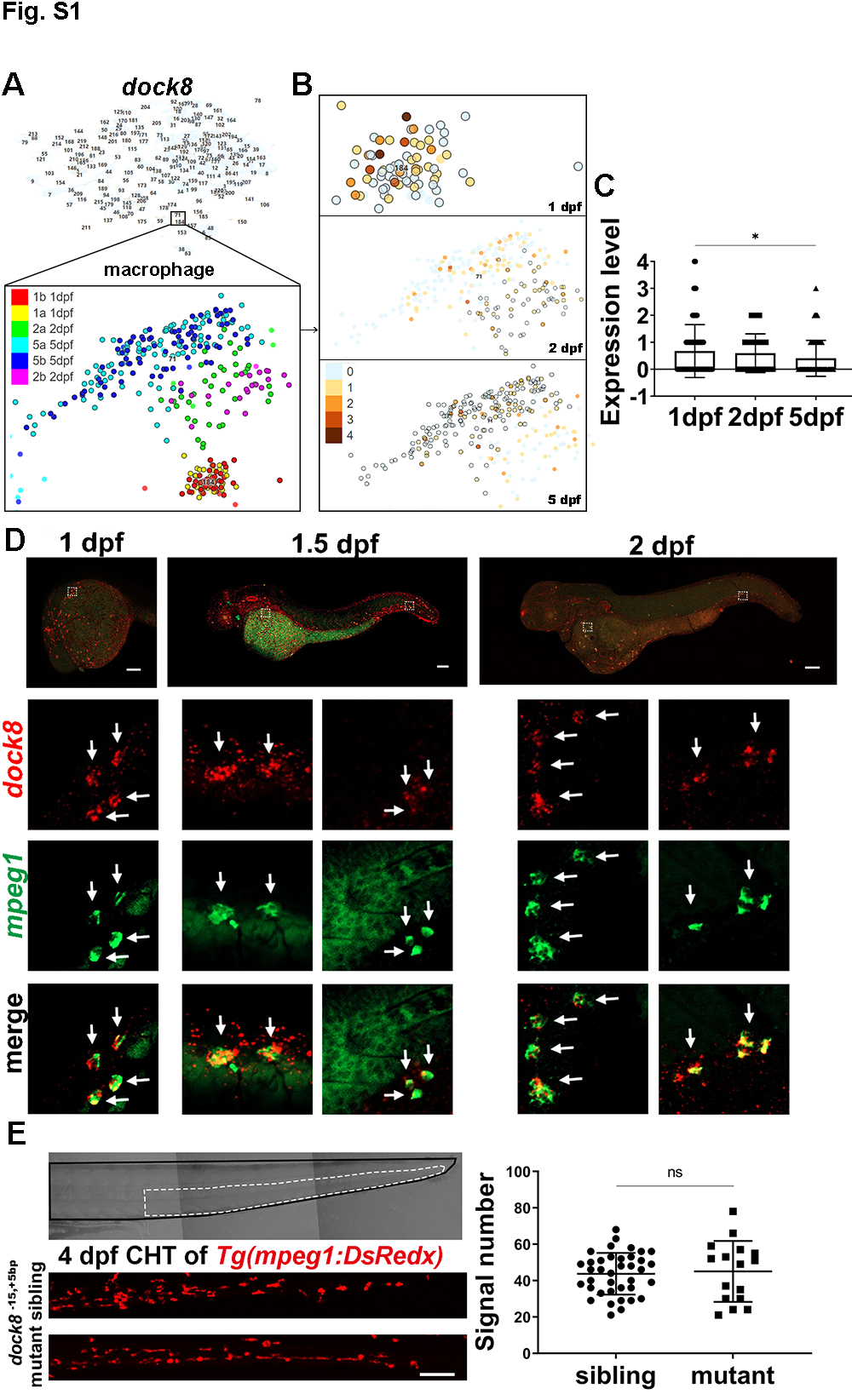

Supplement: Supplementary file 2 — dock8 is expressed in macrophages in early larval stage [file 41420_2022_1155_MOESM2_ESM.tif]

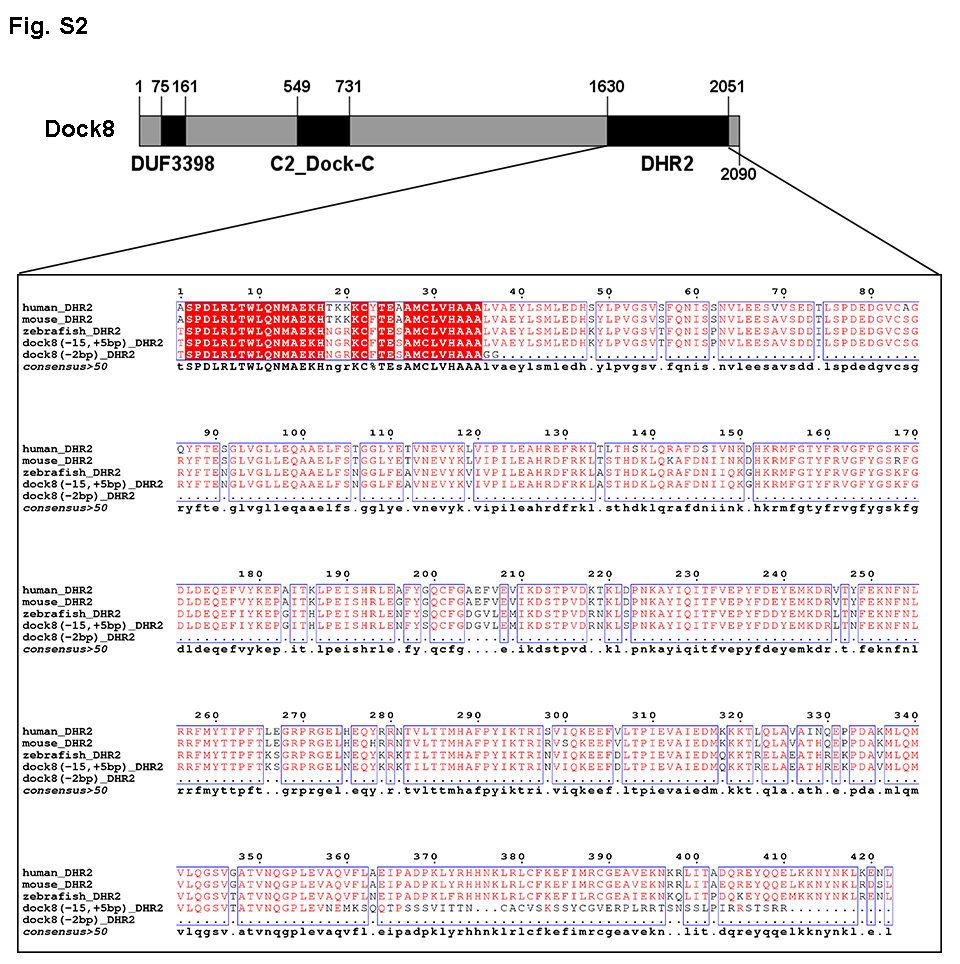

Supplement: Supplementary file 3 — Amino acid alignment of the DHR2 domain in Dock8 from human, mouse, zebrafish and dock8 mutant [file 41420_2022_1155_MOESM3_ESM.tif]

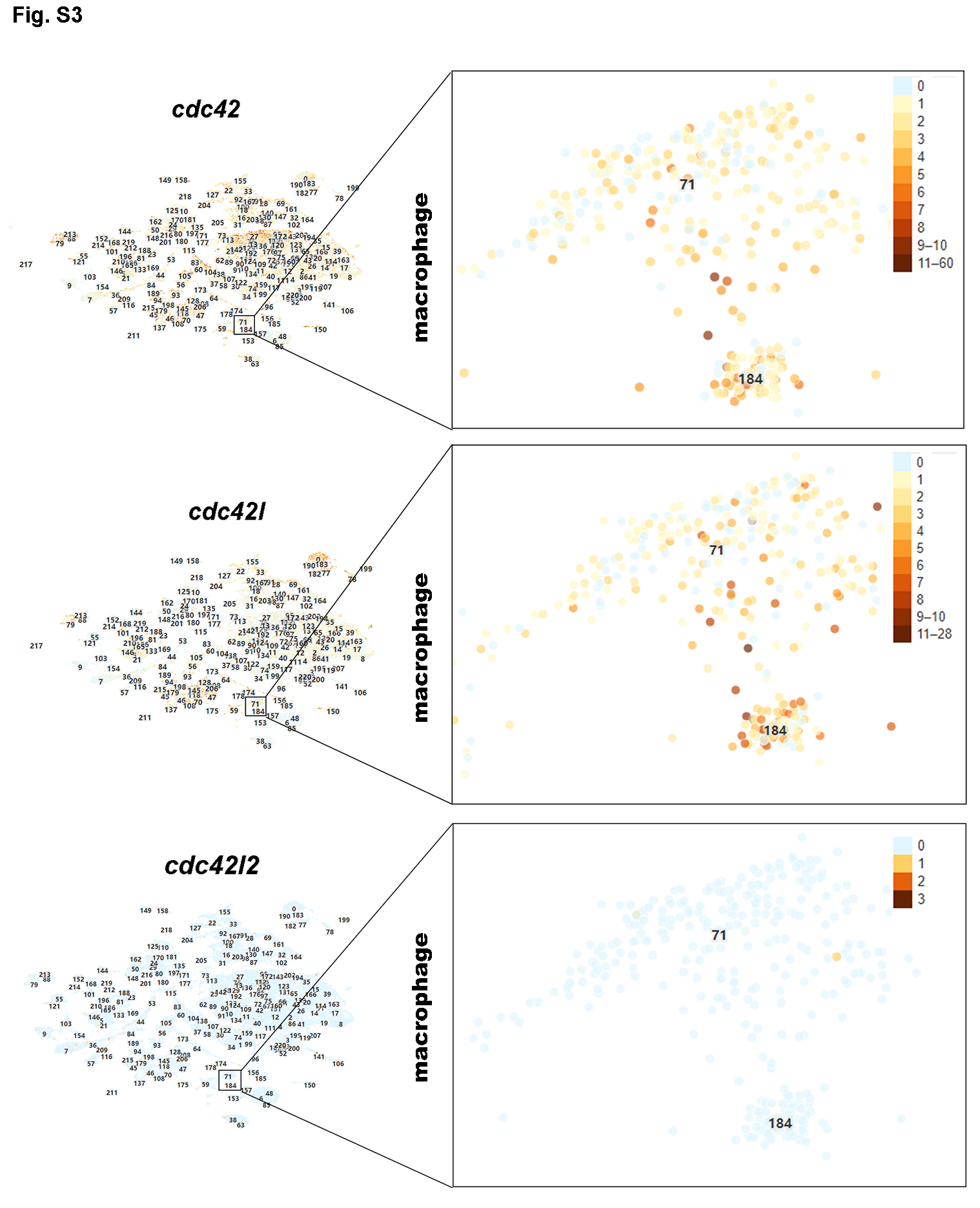

Supplement: Supplementary file 4 — cdc42, cdc42l and cdc42l2 expression in macrophages [file 41420_2022_1155_MOESM4_ESM.tif]

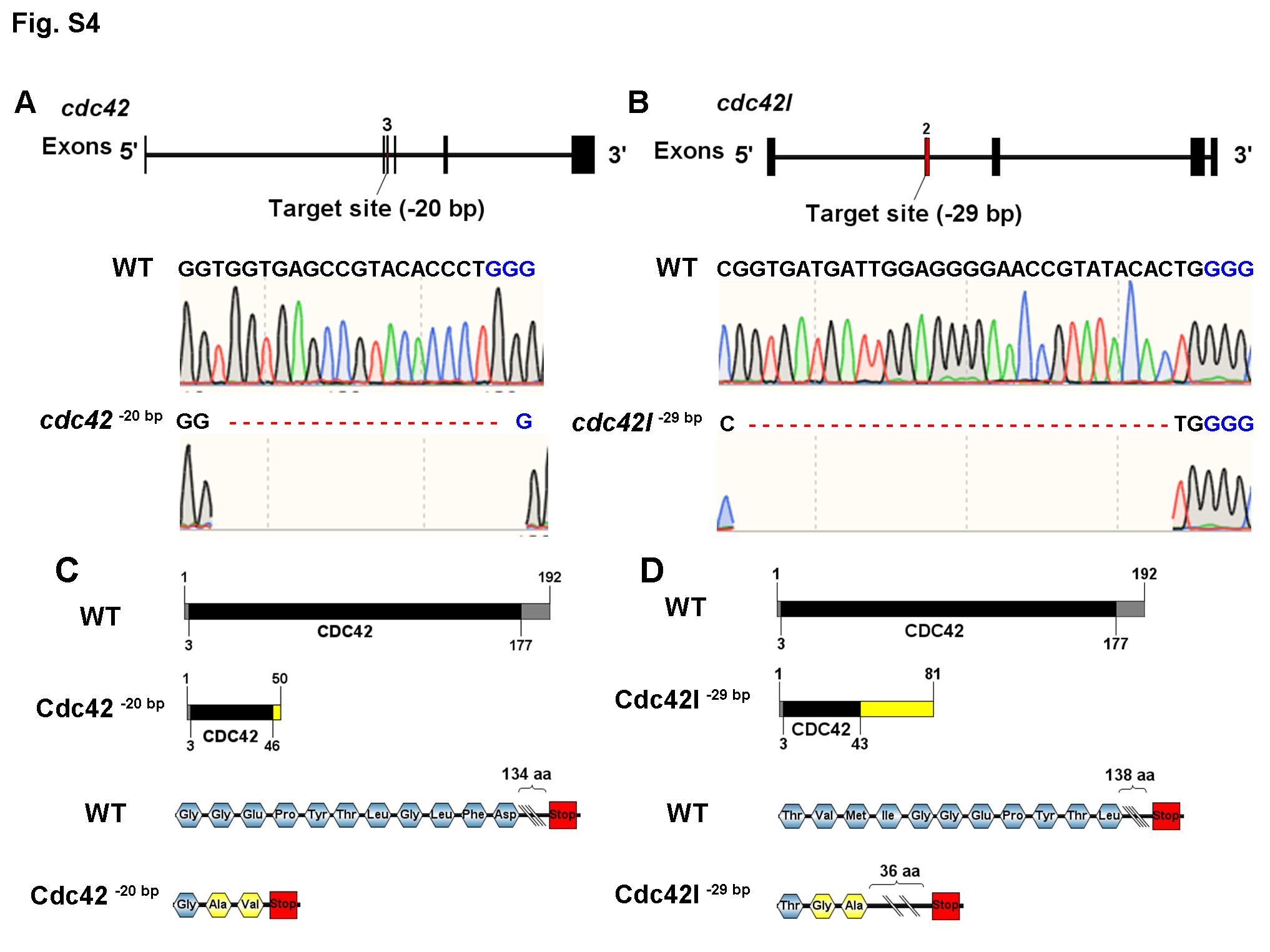

Supplement: Supplementary file 5 — Generation of the cdc42 and cdc42l mutants by CRISPR/Cas9 [file 41420_2022_1155_MOESM5_ESM.tif]

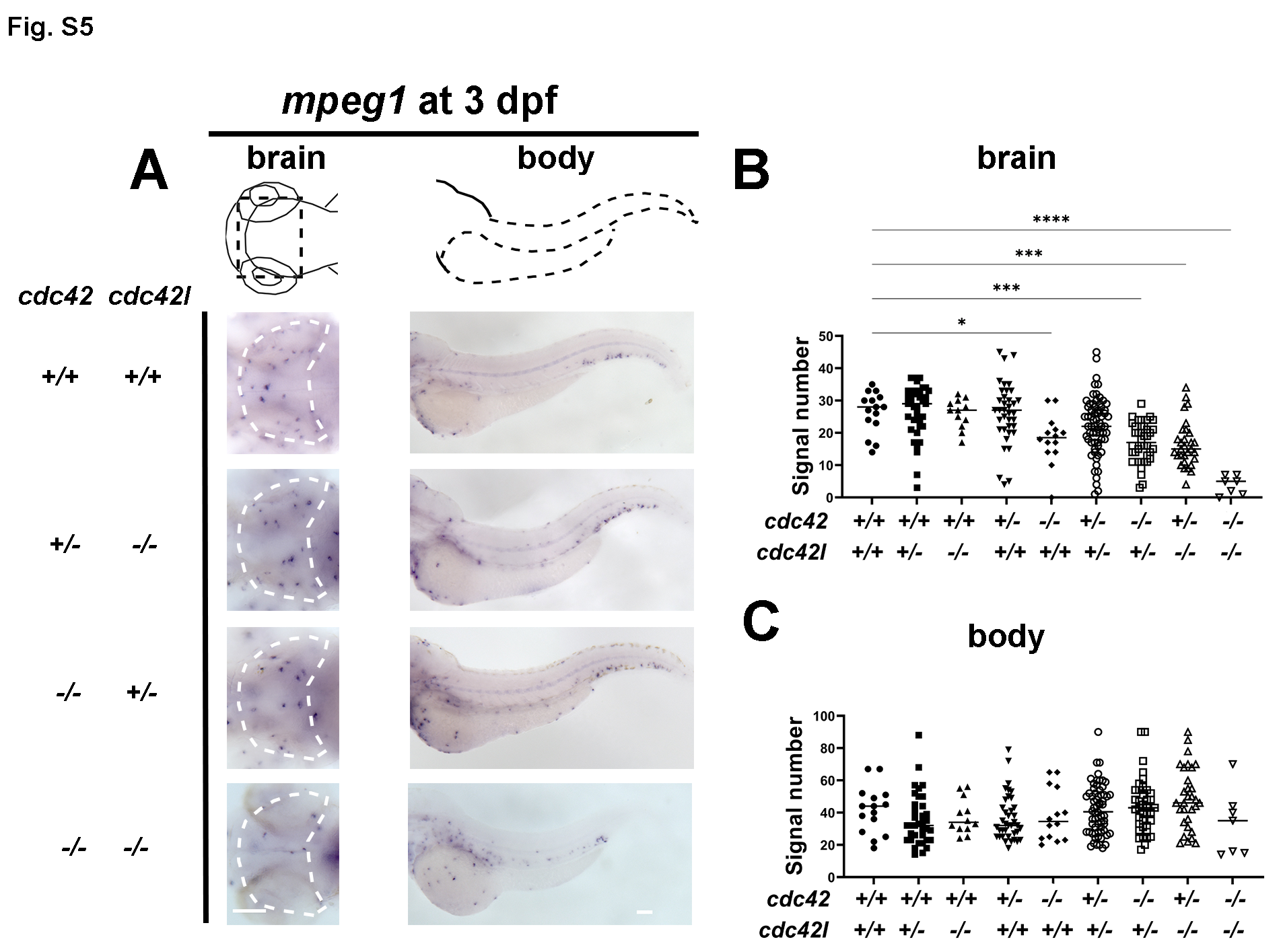

Supplement: Supplementary file 6 — Microglia deficiency in cdc42 and cdc42l mutants [file 41420_2022_1155_MOESM6_ESM.tif]
